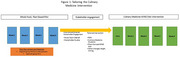# Implementing a culinary medicine intervention to promote the MIND diet in older adults with type 2 diabetes at risk for Alzheimer's disease and related dementias

**DOI:** 10.1002/alz70860_107234

**Published:** 2025-12-23

**Authors:** Mirnova E Ceide, Sophie Anfang, Atul Bhattiprolu, Isabelle Ilan, Akhil Upad, Jessica Shapiro, Samantha Johnson, Paola Ogando, Mairim Melecio‐Vazquez, Jessica L Zwerling, David Lounsbury

**Affiliations:** ^1^ Albert Einstein College of Medicine, New York, NY, USA; ^2^ Montefiore Einstein, Bronx, NY, USA; ^3^ Albert Einstein College of Medicine and Montefiore Medical Center, Bronx, NY, USA; ^4^ Independent Contractor, New York, NY, USA; ^5^ Albert Einstein College of Medicine, Bronx, NY, USA

## Abstract

**Background:**

Type 2 Diabetes (T2DM) is associated with a 60% increased risk of developing any dementia. The 2024 Lancet Commission on Dementia Prevention highlighted diabetes amongst 14 modifiable risk factors. A plant forward dietary pattern such as the MIND Diet has been linked to better glycemic control and improved cognitive function. However, African American and Hispanic older adults diagnosed with hypertension and/or diabetes are 77% less likely to meet the daily recommendation for fruits and vegetables then White counterparts. The aim of this study is to tailor and implement a culinary medicine intervention to promote the MIND diet amongst racially/ethnically diverse older adults with diabetes at risk for Alzheimer's disease and related dementias and their care partners.

**Methods:**

In 2024, we recruited 11 Non‐Hispanic Black and Hispanic older adults with T2DM to participate in two English and Spanish stakeholder studios. They provided feedback on the study aims and design. Utilizing their feedback and our prior experience conducting a Whole Food, Plant Based nutrition education pilot in a similar population, we implemented a nutrition education and culinary medicine workshop series.

**Results:**

Overall, the participants liked the following aspects of the proposed study: the opportunity to learn how to manage diabetes, the chance to address fears about eating certain foods, the culinary demonstration, and the free transportation. They disliked certain aspects of our prior nutrition pilot including: requiring a care partner, length of the classes (2 hours), evening hours, lack of animal‐based protein options, and multiple team members contacting them. Based on this feedback: we adapted the study to make care partners optional, moved the classes to lunch time, shifted to a plant forward MIND dietary pattern, and created a workbook with photos and names of all the team members. We added a registered dietician with experience working with older adults with multimorbidity and medical students, certified in culinary medicine to conduct the workshops. Thus far these changes have increased recruitment and retention threefold.

**Conclusions:**

In this study, we successfully utilized stakeholder engagement strategies including stakeholder studios to tailor and implement a MIND diet intervention in older adults with T2DM.